# Ten-year follow-up of renal adenomatosis with magnetic resonance imaging: a case report

**DOI:** 10.1186/s13256-022-03394-8

**Published:** 2022-04-22

**Authors:** Yi-Chen Chou, Wen-Ying Lee, Steven K. Huang, Reng-Hong Wu, Yu-Ting Kuo

**Affiliations:** 1 Department of Medical Imaging, Chi Mei Medical Center, 901, Chung-Hwa Road, Yung-Kang, Tainan, 710 Taiwan; 2 Department of Pathology, Chi Mei Medical Center, Tainan, Taiwan; 3 Department of Pathology, College of Medicine, Taipei Medical University, Taipei, Taiwan; 4 Department of Urology, Chi Mei Medical Center, Tainan, Taiwan; 5 Department of Medical Imaging, Kaohsiung Medical University Hospital, Kaohsiung, Taiwan; 6 Department of Radiology, Faculty of Medicine, College of Medicine, Kaohsiung Medical University, Kaohsiung, Taiwan

**Keywords:** Renal adenomatosis, Renal cell carcinoma, Magnetic resonance imaging, Case report

## Abstract

**Background:**

Renal adenomatosis is a rare disease that presents as multiple papillary adenomas in the bilateral kidneys. Moreover, papillary adenoma is considered a precursor to papillary renal cell carcinoma. Therefore, patients with renal adenomatosis may have higher risk of developing malignancy than patients without this benign condition.

**Case presentation:**

We present the case of a 62-year-old Asian woman with past history of papillary thyroid cancer. She underwent contrast-enhanced magnetic resonance imaging of the abdomen to screen for metastasis in 2010 and was followed up with computed tomography or magnetic resonance imaging annually. She was found to have a right renal tumor on computed tomography and underwent partial nephrectomy. The pathological diagnosis of the right renal tumor was angiomyolipoma. Renal adenomatosis was also histologically confirmed in the renal parenchyma adjacent to the angiomyolipoma. In this case report, we demonstrate the natural course of renal adenomatosis over 10 years using imaging studies. The benign tumors gradually progressed during the follow-up period. Larger tumor sizes and more hypoenhanced nodules in the bilateral kidneys were observed on follow-up computed tomography and magnetic resonance imaging.

**Conclusions:**

Due to its malignant potential, the clinical course of renal adenomatosis must be monitored. We present the natural course of renal adenomatosis with magnetic resonance imaging during a 10-year follow-up period.

## Background

Renal papillary adenoma is a benign epithelial tumor of the kidney with the following pathological features: unencapsulated tumor with papillary, tubulopapillary, or tubular architecture, low nuclear-to-cytoplasmic ratio, and diameter less than 15 mm. Renal adenomatosis is characterized by multiple (usually more than five) adenomas in one kidney [1]. Syrjanen *et al.* reported the first case of renal adenomatosis in 1978 [2], and since then, only a few cases have been reported. The incidence of papillary adenoma increases with age. Patients with renal adenomatosis might be diagnosed with glomerulosclerosis or chronic renal damage [3–5]. Papillary adenoma is considered a precursor lesion of papillary renal cell carcinoma because it shares similar immunohistochemical profiles and genetics, as reported in recent studies [6–8]. There is no unanimously accepted standard treatment for this disease. We report a particular case of renal adenomatosis with a series of imaging studies over a 10-year follow-up period.

## Case presentation

**Fig. 1:**
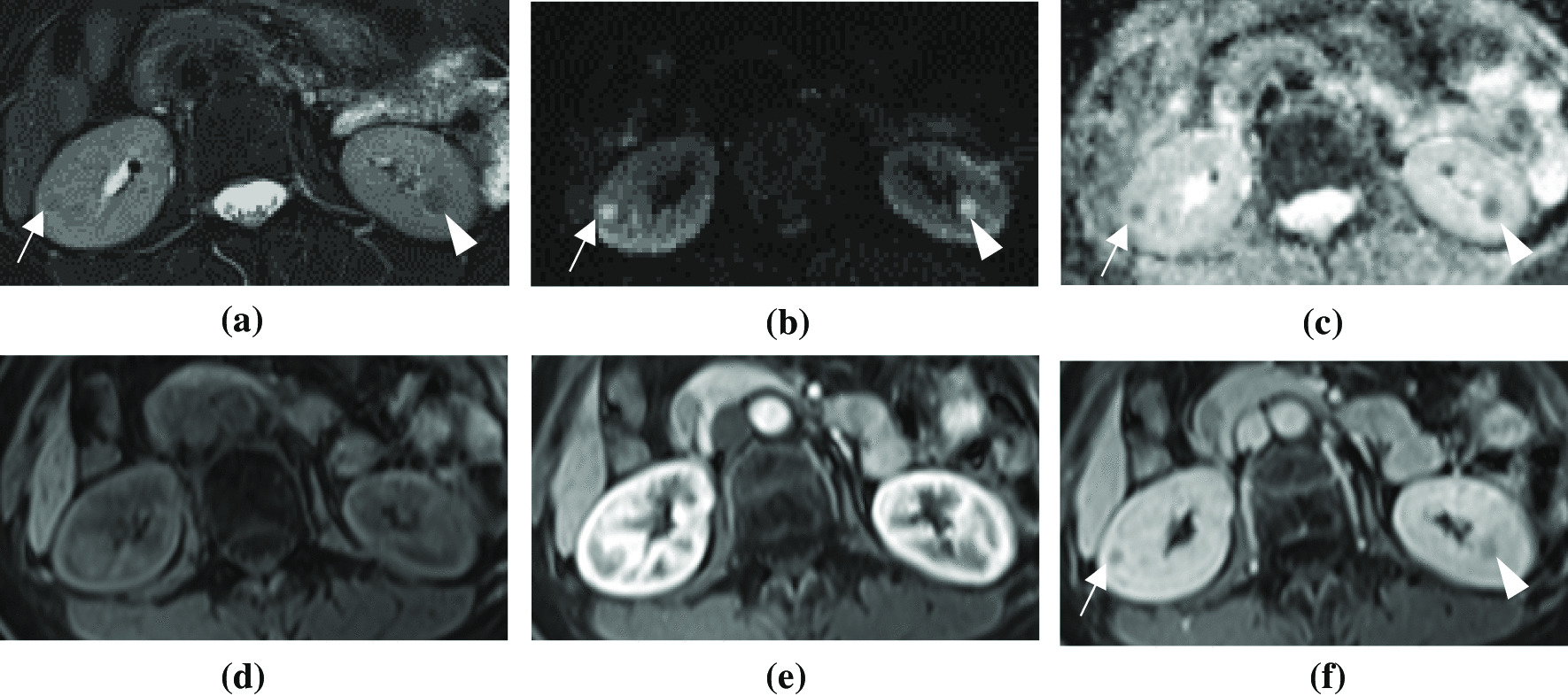
Abdominal MRI with contrast enhancement performed in January 2010. **a** On axial fat-saturated T2-weighted images, papillary adenomas present as well-defined hypointense nodules (arrow and arrowhead) in the bilateral renal parenchyma. **b**, **c** Papillary adenomas present high signal intensity (arrow and arrowhead) on axial diffusion-weighted (*b*-value: 800) images and low signal intensity (arrow and arrowhead) on the corresponding apparent diffusion coefficient maps, suggesting diffusion restriction. **d**, **e**, **f** On axial dynamic contrast-enhanced T1-weighted images with fat suppression, papillary adenomas present isointensity in the renal parenchyma on unenhanced images and relatively poor contrast enhancement in the arterial and venous phases (arrow and arrowhead)

**Fig. 2:**
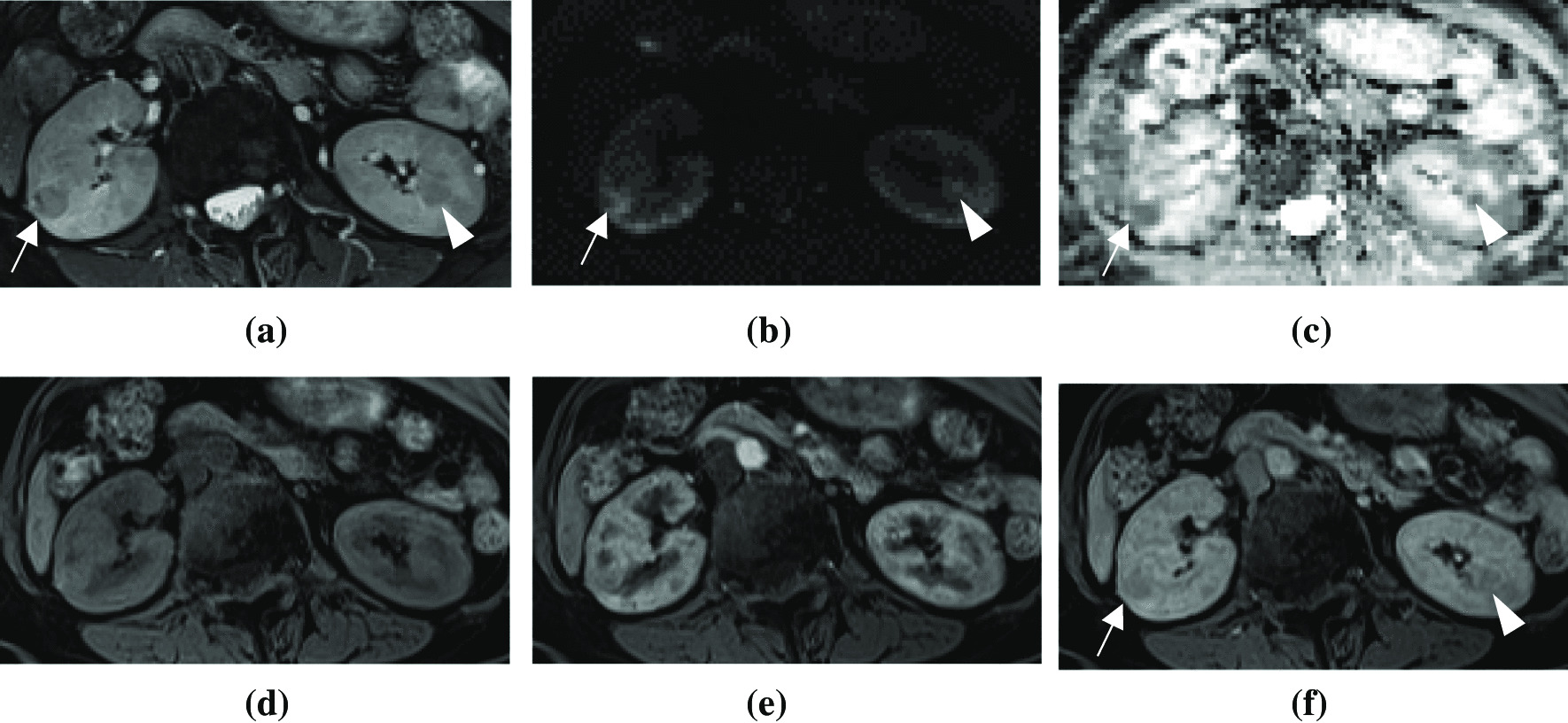
Abdominal MRI performed in August 2020. **a** On axial T2-weighted images, multiple papillary adenomas were observed in the renal cortices of the bilateral kidneys (arrow and arrowhead). Progression of the tumors was evident based on the increased tumor size and tumor number. **b**, **c** On axial diffusion-weighted images and apparent diffusion coefficient maps, more renal adenomas were noted in the bilateral kidneys with diffusion restriction (arrow and arrowhead). The imaging features remained consistent with those seen 10 years earlier. **d**, **e**, **f** On axial dynamic contrast-enhanced T1-weighted images with fat suppression, papillary adenomas present isointensity relative to the renal parenchyma on unenhanced images and relatively poor contrast enhancement in the arterial and venous phases (arrow and arrowhead). These imaging characteristics remained consistent with those seen 10 years earlier

A 62-year-old Asian woman with hypertension was under medical control with β-adrenergic blocking agents and calcium channel blockers for approximately 10 years. Hypertension was also noted in her family history. She underwent a thyroidectomy for treatment of papillary thyroid cancer in 2004. After the operation, she regularly took levothyroxine to maintain her thyroid hormone level. She denied smoking or consuming alcohol. She had a daughter and entered menopausal status when she was 49 years old. She underwent screening with contrast-enhanced magnetic resonance imaging (MRI) of the abdomen in 2010 (Fig. 1). This MRI study revealed multiple nodules in the bilateral renal cortices, with mild hypointensity on T2-weighted (T2W) images and isointensity of the adjacent renal cortex on T1-weighted (T1W) images. Diffusion restriction on the apparent diffusion coefficient (ADC) map and hyperintensity on diffusion-weighted images (DWIs) were also seen. Relatively poor enhancement on contrast-enhanced T1W images was observed. Ten years later, follow-up MRI findings in 2020 (Fig. 2) revealed a higher tumor number and larger lesions than seen in previous studies in 2010. In 2010, most of the tumors were smaller than 10 mm, while in 2020, several tumors were larger than 10 mm. Otherwise, these renal nodules presented with imaging features similar to those observed 10 years earlier. Biological markers of kidney function (such as serum creatinine, blood urea nitrogen, and urine analysis) were within normal limits (Tables 1 and 2). However, the tumor marker CA 19-9 level increased progressively over the 10-year period (from 228.2 U/mL in 2010 to 654.9 U/mL in 2020), while no apparent tumors were noted in the hepatobiliary system or pancreas.

**Table 1:** Blood tests in 2010, 2018, and 2020

	2010	2018	2020	Reference
CBC				
WBC (×10^6^/µL)	5.3	6.0	7.3	3.2–9.2
RBC (×10^6^/µL)	3.83	3.64	4.00	3.7–4.9
Platelets (×10^3^/µL)	163	192	187	150–400
GOT (IU/L)	24	20	22	5–34
GPT (IU/L)	27	13	19	2–40
BUN (mg/dL)	19	17	20	6–20
Creatinine (mg/dL)	0.6	0.85	0.75	0.57–1.11
CA 19-9 (U/mL)	187	502	798	< 37
T4 (ng/dL)	1.28	6.4	7.1	4.5–12.5
TSH (µIU/mL)	0.12	5.89	0.51	0.25–4.0

*CBC* complete blood count, *WBC* white blood cell count, *RBC* red blood cell count, *GOT* glutamic oxaloacetic transaminase, *GPT* glutamic pyruvic transaminase, *BUN* blood urea nitrogen, *CA 19-9* carbohydrate antigen 19-9, *TSH* thyroid-stimulating hormone

**Table 2:** Urinary analysis in 2010, 2018, and 2020

	2010	2018	2020	Reference
Appearance	Clear	Clear	Clear	Clear
Sediment-WBC (/HPF)	<1	<1	1–4	0–5
Sediment-RBC (/HPF)	<1	<1	1–4	0–2
Sediment-Bacilli (/HPF)	–	–	–	–
Leukocyte esterase	–	–	+/–	–

*WBC* white blood cell count, *RBC* red blood cell count, *HPF* high-power field

**Fig. 3:**
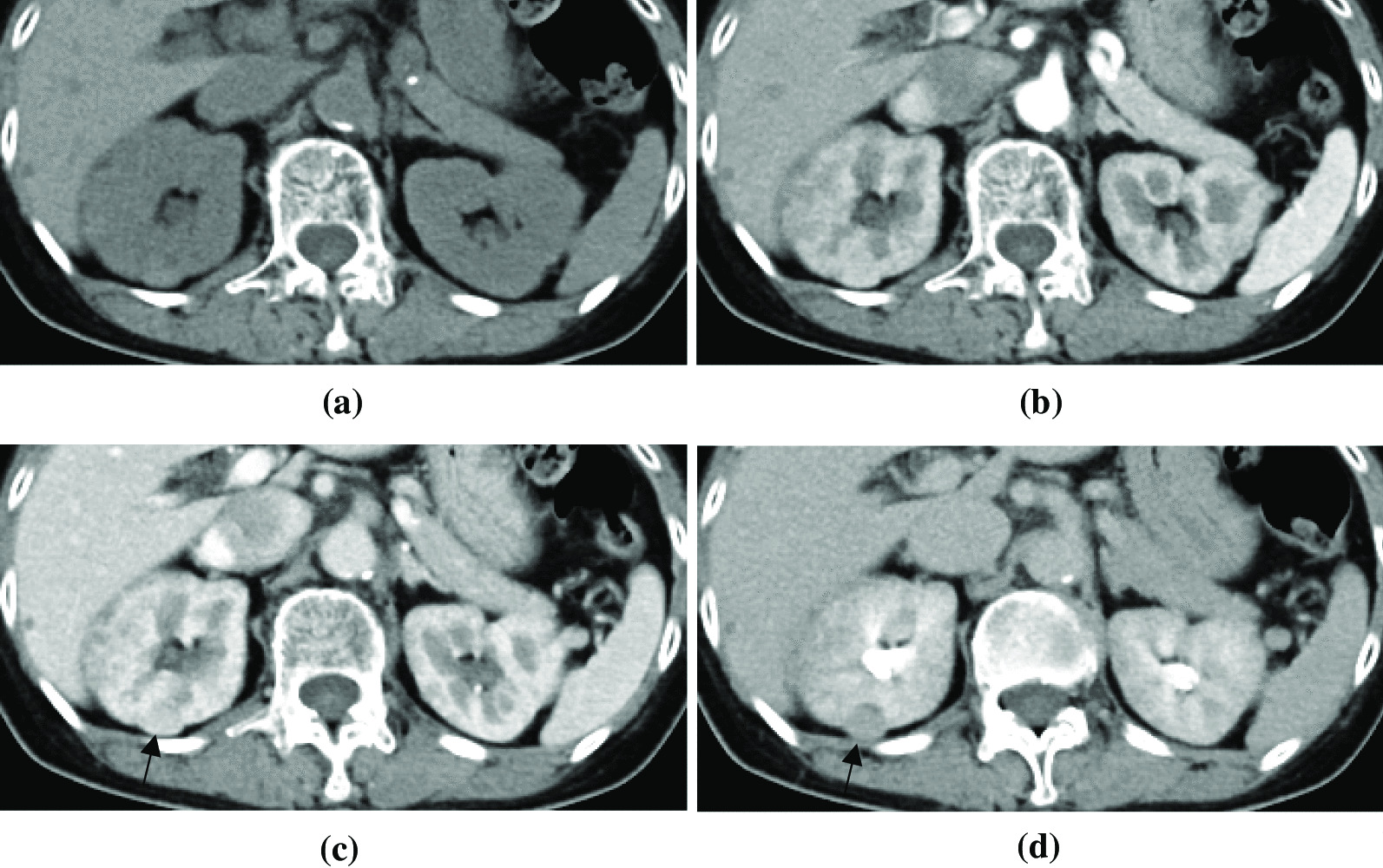
Iodine-enhanced CT images acquired in October 2018. **a** On unenhanced CT images, a slightly hyperdense and exophytic-growing nodule was observed (pathological analysis showed epithelioid angiomyolipoma) at the posterior aspect of the right kidney. **b** In arterial-phase CT images, the epithelioid angiomyolipoma was significantly enhanced. In addition, there were multiple small renal nodules (pathological analysis showed papillary adenomas) with poor enhancement in the bilateral kidneys. **c**, **d** On nephrographic and excretory-phase computed tomography images, the epithelioid angiomyolipoma (black arrow) was hypodense relative to the adjacent renal cortex. The papillary adenomas were iso- to hypodense in the renal cortex in the excretory phase

**Fig. 4:**
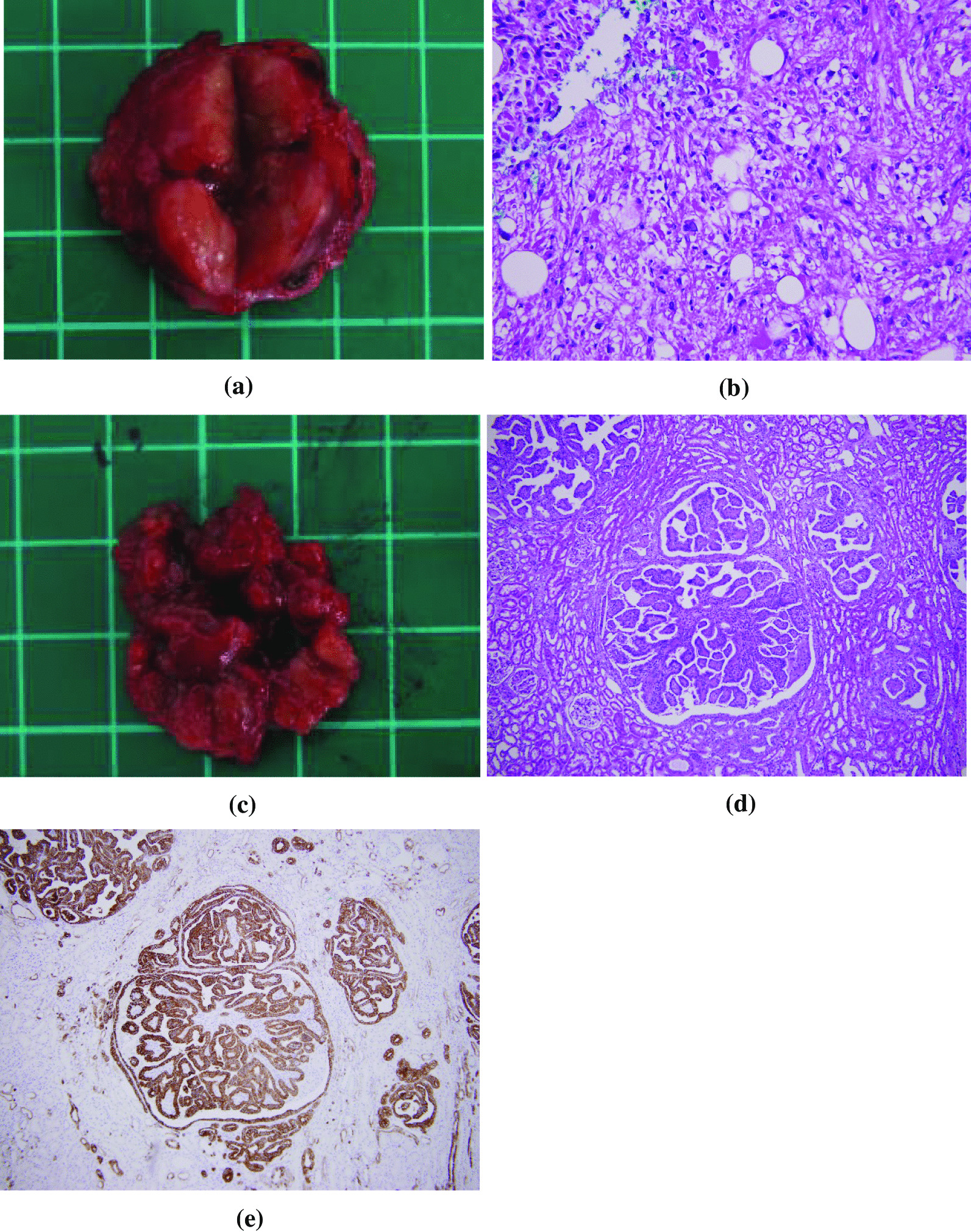
Pathology of renal adenomatosis and epithelioid angiomyolipoma obtained during surgery in November 2018. **a** The gross specimen from the right partial nephrectomy showed a well-defined renal tumor with a mildly pale appearance. **b** Photomicrographs show the tumor, and there were predominant myomatous elements admixing with tortuous vessels and some adipose elements (hematoxylin–eosin stain; original magnification, ×400). **c** The gross specimen from the right partial nephrectomy was peritumoral soft tissue showing multiple small nodular-like lesions with a mildly pale appearance. **d** The photomicrograph shows renal adenoma and nodules with papillary architecture lined by epithelial cells with a low nuclear-to-cytoplasmic ratio (hematoxylin-eosin stain; original magnification, ×100). **e** The photomicrograph of the renal adenomas showed positive immunohistochemical staining for cytokeratin 7

A renal tumor was noted in the right kidney on a follow-up contrast-enhanced computed tomography (CT) scan in 2018 (Fig. 3), which showed an enhancing pattern that differed from that of the other renal nodules. In addition, there were multiple hypoenhanced nodules in both kidneys in the parenchymal phase. Because the possibility of a malignant tumor could not be excluded, the patient subsequently underwent right partial nephrectomy for tumor resection in November 2018. During admission, there were no abnormal findings on physical or neurological examinations (her sclera was anicteric, her conjunctiva was pink, no neck mass, clear breathing sounds, no heart murmur, soft abdominal wall without striae, normal muscle power, and deep tendon reflex), and her vital signs were also stable (Table 3). She continued taking antihypertensive medication and levothyroxine during her hospital stay. The renal tumor was a well-defined tumor with a predominant myomatous element admixing with tortuous vessels and some adipose elements, which are pathological characteristic features of epithelioid angiomyolipoma. Furthermore, some large and unusual hyperchromatic nuclei without evident mitotic activity were also noted. Immunohistochemically, the tumor cells were positive for melan-A protein and smooth muscle actin staining (Fig. 4a, b).

**Table 3:** Vital signs in 2010, 2018, and 2020

	2010	2018	2020
Pulse rate (/min)	71	60	67
Blood pressure (mmHg)	135/77	124/68	143/76
Body temperature (°C)	37.0	36.4	36.5
Respiration rate (/min)	18	19	18

Furthermore, in addition to angiomyolipoma, there were multiple small renal tumors in the adjacent renal parenchyma, which were considered to be papillary adenomas (Fig. 4c). Pathological analysis of peritumoral tissue from the partial nephrectomy specimen showed multiple unencapsulated proliferative epithelial nodules with papillary, tubular, or tubulopapillary configurations (Fig. 4d). The tumor cells had low-grade nuclei. The tumors were as large as 1 cm. Immunohistochemically, the tumor cells were positive for cytokeratin 7 (CK7) (Fig. 4e) and focally positive for α-methylacyl-coenzyme A racemase (AMACR, also called p504s) but negative for thyroid transcription factor-1. Therefore, we excluded the possibility of metastasis with a thyroid origin. The multiple tumors showed a morphology and immunophenotype similar to those of papillary renal cell carcinoma; however, the size of these papillary tumors did not exceed 15 mm. Therefore, a diagnosis of renal adenomatosis was established.

Due to the malignant potential of renal adenomatosis, tumor biopsy was performed under ultrasound guidance in September 2020. The pathological analysis revealed no malignant features and no increase in the nuclear-to-cytoplasmic ratio.

## Discussion

The patient was a 62-year-old female with hypertension and papillary thyroid cancer. She was incidentally found to have renal adenomatosis and underwent MRI follow-up for 10 years. For the first time, we demonstrate the natural course of renal adenomatosis using MRI.

Renal papillary adenoma is a benign tumor that is reported in 7% of kidney resections performed for other tumors, most commonly for renal cell carcinoma, showing an incidence of 19% in an autopsy study [3, 7]. Renal adenomatosis is characterized by five or more adenomas in one kidney. The prevalence of renal adenomatosis has not been previously reported. The first case of renal adenomatosis was reported by Syrjanen in 1979 [2], and only 16 cases of renal adenomatosis have been reported to date. The incidence of papillary adenoma increases with age, glomerulosclerosis, or chronic renal damage [3–5]. According to Kim *et al.* [9], renal papillary adenoma can also be observed in patients with acquired cystic disease or hereditary papillary renal cancer.

Pathologically, renal papillary adenoma and papillary renal cell carcinoma can be divided into four subgroups (types A, B, C, and D) and two subgroups (types 1 and 2), respectively. Type A and D renal papillary adenomas have similar histology and genetic features to type 1 and 2 papillary renal cell carcinoma [3]. These findings are consistent with those reported in a study conducted by Brunelli *et al.* They found similar chromosomal changes between renal papillary adenoma and papillary renal cell carcinoma [7, 8]. Similar immunohistochemical markers (CK7 and AMACR) have also been reported between papillary adenoma and papillary renal cell carcinoma [7, 9, 10]. In this case, we also observed positive immunohistochemical staining for CK7 and AMACR.

Renal adenomatosis is rarely diagnosed preoperatively, not only because of its low prevalence but also because of radiologists’ unfamiliarity with its imaging features. To the best of our knowledge, this is the first report to demonstrate the natural course of renal adenomatosis with a series of imaging studies over a 10-year follow-up period.

Some experts consider that papillary adenoma and papillary renal cell carcinoma have a similar disease course. In the 2016 World Health Organization (WHO) classification system, the threshold for papillary renal adenoma is 15 mm in size, while the corresponding threshold was 5 mm in the 2004 WHO classification system. The reason for this is the low probability of metastasis when a renal tumor is smaller than 20 mm [11]. Additionally, Thompson *et al.* reported that the risk of metastasis may be negligible if the tumor is smaller than 30 mm after the patient undergoes nephrectomy [12]. Renal papillary adenoma certainly has malignant potential. However, apart from the size criterion, how to determine whether an adenoma will progress to papillary renal cell carcinoma remains unknown. In the present case, the adenoma showed slow progression over 10 years without evidence of metastasis. Currently, there is no definite treatment for renal adenomatosis. The benefits of prophylactic surgery, such as bilateral nephrectomy, remain unclear.

Several other diseases need to be differentiated from renal adenomatosis on imaging studies because they also demonstrate small hypoenhanced nodules in bilateral renal parenchyma. For example, renal tuberculosis should be considered. Tuberculosis usually initially spreads in the medullary portion of the kidney and is often associated with collecting system diseases, such as infundibular stenosis with caliectasis, ureteral stricture, or ureteral wall thickening. Some cortical granulomas coalesce with calcifications. In immunocompromised patients, miliary tuberculosis may be observed, appearing as multiple hypoenhanced nodules in the bilateral kidneys that are usually smaller than 3 mm in diameter and in the renal cortex [13]. On enhanced CT images, cortical granulomas are hypoattenuated relative to the adjacent renal cortex [14].

Renal lymphoma can present as a solitary renal nodule, multiple bilateral renal nodules, soft tissue in the perinephric space, a direct extension from retroperitoneal adenopathy, or infiltration lesions in the bilateral kidneys [15]. The size of lymphomas usually varies, but lymphomas larger than 15 mm in diameter are more common. On CT images, renal lymphoma is usually more hypoenhanced than the adjacent renal cortex but is gradually enhanced in the corticomedullary phase. On MRI, renal lymphoma can be hypointense on T1W images and slightly more hypo- or isointense than the adjacent normal renal cortex on T2W images, with gradual contrast enhancement on postcontrast T1W images. Lymphomas usually show diffusion restriction on DWI and ADC maps. Renal lymphoma usually shows marked fluorodeoxyglucose (FDG) uptake on positron emission tomography images [16].

Immunoglobulin G4 (IgG4)-related disease is a systemic disease, and the most commonly affected organ is the pancreas, resulting in IgG4-related autoimmune pancreatitis. Isolated IgG4-related renal disease is rare; only 3% of patients have renal lesions without involvement of other organs [17]. Five IgG4-related renal disease patterns have been described, and bilateral round- or wedge-shaped peripheral cortical lesions are the most common type [18]. On CT images, the bilateral round renal nodules in the peripheral cortex show similar attenuation to the renal parenchyma and hypoattenuation relative to the renal parenchyma on enhanced images. IgG4-related renal nodules are hypointense on T1W and T2W MR images and mildly enhanced on postcontrast T1W MR images [19].

## Conclusions

We report a case with a series of follow-up CT and MRI studies over 10 years that demonstrates the natural course and behavior of renal adenomatosis. Awareness of the imaging features on CT and MRI might help radiologists diagnose renal adenomatosis before surgery.

## Data Availability

All data generated or analyzed during this study are included in this published article.

## Abbreviations

MRI: Magnetic resonance imaging
CT: Computed tomography
CK7: Cytokeratin 7
AMACR: α-Methylacyl-coenzyme A racemase
T1W: T1-weighted
T2W: T2-weighted
ADC: Apparent diffusion coefficient
DWI: Diffusion-weighted image
FDG: Fluorodeoxyglucose
IgG4: Immunoglobulin G4
WHO: World Health Organization
